# A temperature-controlled cold-gas humidifier and its application to protein crystals with the humid-air and glue-coating method

**DOI:** 10.1107/S1600576719006435

**Published:** 2019-06-14

**Authors:** Seiki Baba, Atsuhiro Shimada, Nobuhiro Mizuno, Junpei Baba, Hideo Ago, Masaki Yamamoto, Takashi Kumasaka

**Affiliations:** a Japan Synchrotron Radiation Research Institute, 1-1-1 Kouto, Sayo-cho, Sayo-gun, Hyogo, 679-5198, Japan; b Picobiology Institute, Graduate School of Life Science, University of Hyogo, 3-2-1 Kouto, Kamigori-cho, Ako-gun, Hyogo, Japan; c RIKEN SPring-8 Center, 1-1-1 Kouto, Sayo-cho, Sayo-gun, Hyogo, 679-5148, Japan

**Keywords:** temperature control, humidity control, X-ray diffraction, protein crystals, glue coating

## Abstract

The room-temperature experiment has been revisited for macromolecular crystallography. Despite being limited by radiation damage, such experiments reveal structural differences depending on temperature, and it is expected that they will be able to probe structures that are physiologically alive. For such experiments, the humid-air and glue-coating (HAG) method for humidity-controlled experiments is proposed. The HAG method improves the stability of most crystals in capillary-free experiments and is applicable at both cryogenic and ambient temperatures. To expand the thermal versatility of the HAG method, a new humidifier and a protein-crystal-handling workbench have been developed. The devices provide temperatures down to 4°C and successfully maintain growth at that temperature of bovine cytochrome c oxidase crystals, which are highly sensitive to temperature variation. Hence, the humidifier and protein-crystal-handling workbench have proved useful for temperature-sensitive samples and will help reveal temperature-dependent variations in protein structures.

## Abstract 2

A new temperature-controllable humidifier for X-ray diffraction has been developed. It is shown that the humidifier can successfully maintain protein crystal growth at a temperature lower than room temperature.

## 1. Introduction   

X-ray free-electron lasers (XFELs) are used in macromolecular crystallography to overcome difficulties in radiation-damage-free analysis and time-resolved (TR) measurements (Chapman *et al.*, 2011 ▸; Kupitz *et al.*, 2014 ▸; Tenboer *et al.*, 2014 ▸; Keedy *et al.*, 2015 ▸; Schlichting, 2015 ▸; Nango *et al.*, 2016 ▸; Nogly *et al.*, 2016 ▸). Although TR serial crystallography was initially developed with XFELs, it affects synchrotrons too, and increasing use of TR analysis is expected with both XFELs and synchrotrons (Levantino *et al.*, 2015 ▸; Panneels *et al.*, 2015 ▸).

In TR serial crystallography, protein crystal samples are maintained *in situ*, that is, under delicate ambient-temperature conditions. Thus, care should be taken when mounting and delivering such samples. Several types of devices have been developed to accomplish this work. A glass capillary tube (Garman & Schneider, 1997 ▸) is conventionally used as a protein-crystal-mounting tool, and a protein-crystal-fishing cryoloop with a sealed tube (Kalinin *et al.*, 2005 ▸) has been recently introduced. However, in experiments, capillary-free protein crystal mounting is better suited to TR analysis. To initiate a reaction and/or dynamical movement of samples, ‘naked’ samples should be directly subjected to changes in environmental conditions and/or illumination by pump or probe lights.

Humidity-control devices, also known as humidifiers, have been developed to maintain protein crystals *in situ* (Kiefersauer *et al.*, 2000 ▸; Sjögren *et al.*, 2002 ▸; Sanchez-Weatherby *et al.*, 2009 ▸). Such devices were initially used in dehydration experiments to improve protein crystal quality (Awad *et al.*, 2013 ▸; Lobley *et al.*, 2016 ▸). However, the effects of temperature and humidity on protein crystals have been further investigated, including different side-chain conformations at cryogenic and ambient temperatures (Petsko & Tsernoglou, 1979 ▸; Tilton *et al.*, 1992 ▸; Juers & Matthews, 2004 ▸; Fraser *et al.*, 2009 ▸; Keedy *et al.*, 2014 ▸; Fischer *et al.*, 2015 ▸), temperature-dependent structures under non-cryogenic conditions providing information about protein dynamics (Keedy *et al.*, 2015 ▸), structural variation in active-site residues affected by humidity and temperature (Atakisi *et al.*, 2018 ▸), and the importance of humidity control to maintain crystal quality during protein crystal manipulation (Farley *et al.*, 2014 ▸).

Despite the easy availability of humidifiers, their versatility is limited because most protein crystals are extremely sensitive to changes in humidity, even when using elaborate humidifiers. To address this issue, an alternative method of controlling humidity – the humid-air and glue-coating (HAG) method – is used. Here, protein crystals are coated with a polymer hydro­gel (Baba *et al.*, 2013 ▸). Previous studies have demonstrated that the gel buffers the moisture exchange between the protein crystals and humid air, and the glue-coated protein crystals maintain their quality under humidity control more easily than uncoated protein crystals (Baba *et al.*, 2013 ▸; Mazzorana *et al.*, 2014 ▸). The HAG method has now been expanded to diverse protein crystals (Matsumoto *et al.*, 2016 ▸; Kaneko *et al.*, 2017 ▸), but it cannot be applied to temperature-sensitive ones because conventional humidifiers can be operated only at room temperature (RT).

In this paper, we describe an adaptation of the HAG method for such protein crystals using a temperature-controlled humidifier and a protein-crystal-handling workbench which enable lower-temperature experiments down to 4°C. We evaluate these devices using protein crystals of bovine heart cytochrome c oxidase (CcO), which are grown at 4°C and are immediately damaged by rising temperature. These devices have already been applied to TR structural analysis of CcO with the SPring-8 Angstrom Compact Free Electron Laser (SACLA) (Shimada *et al.*, 2017 ▸) using serial femtosecond rotation crystallography (Hirata *et al.*, 2014 ▸) at 4°C. In the present work, we confirm the performance of the temperature-controlled humidifier from 4 to 20°C. We also demonstrate that the HAG method can be used in experiments at a non-freezing temperature of 4°C.

## 2. Material and methods   

### 2.1. Humidity-control device for low temperature   

**Figure 1:**
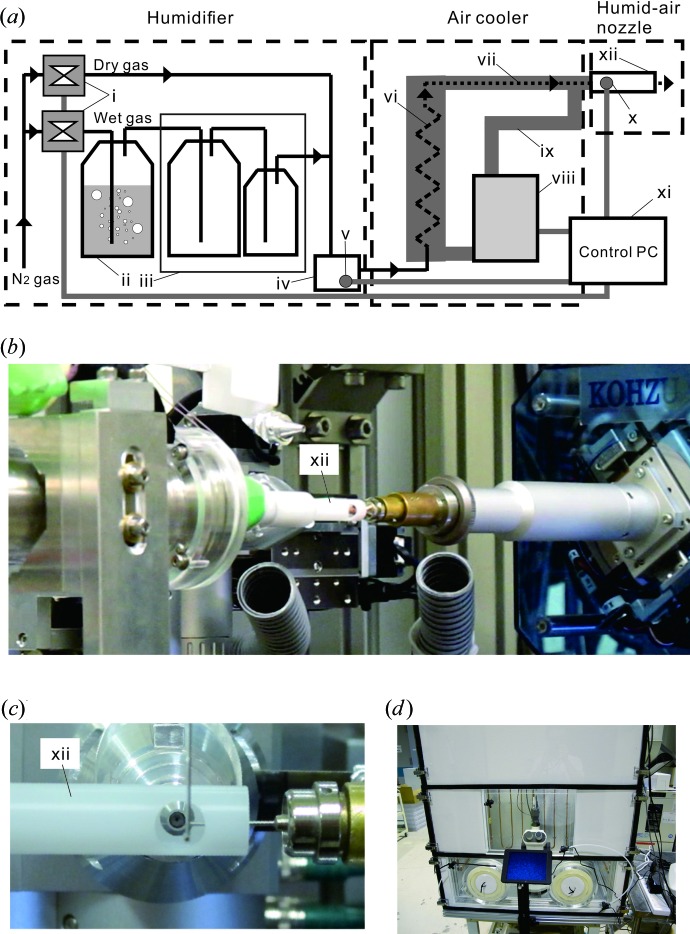
Overview of devices used for the HAG method. (*a*) Design of the TeCH-1 humidifier. This was assembled using the following components: (i) digital mass flow controllers, (ii) temperature-controlled air-bubbling bottle (2 l), (iii) air-cooling bottles (2 l and 500 ml), (iv) air-mixing area, (v) temperature and humidity sensor, (vi) original heat-exchanger unit, (vii) flexible transfer tube, (viii) refrigeration–heating circulator, (ix) coolant line, (x) temperature sensor, (xi) PC to control and monitor connected devices, and (xii) humid-air nozzle. (*b*) View of a diffractometer set up in the humidity-control position. (*c*) Enlarged view of the sample position for insertion of the humid-air nozzle. (*d*) Front view of the TeC-W workbench.

To produce humid air from ambient temperature down to 4°C, our original humidity-control device (Baba *et al.*, 2013 ▸) was created using a newly developed air-cooling unit. The new temperature-controlled humidifier, TeCH-1, is composed of three separate units: a humidifier, an air cooler and a humid-air nozzle [Fig. 1 ▸(*a*)].

The humidifier works at ambient temperature and supplies wet gas to the air cooler. The wet gas is adjusted to a vapour pressure corresponding to the relative humidity (RH) at the desired temperature by mixing dry and saturated humid gases. The saturated humid gas is produced from the dry gas, which is supplied by an N_2_ gas generator (KOFLOC Corporation Ltd, Japan), and bubbled in a water bottle equipped with a rubber heater kept at 28°C. The humid gas is transported and then cooled to room temperature (RT) during transportation before it reaches the air-mixing area. Mixing of the dry and humid gases is regulated using type SEC-N digital mass-flow controllers (Horiba Ltd, Japan). The temperature *t*_1_ (°C) and humidity *h*_1_ (%RH) of the mixed air from the humidifier are measured using a type HYT271 humidity and temperature sensor (IST AG, Switzerland). *t*_1_ is not controlled but is about RT.

The air cooler comprises a heat exchanger and a double-pipe transfer tube. The heat exchanger was assembled with a tube that is passed through once in the shell to even out the coolant’s cooling. The transfer tube is lagged with an insulator and connects the heat exchanger to a humid-air nozzle. The coolant for the heat exchanger is chilled using an F32HE or F50HE refrigerated–heating circulator (JULABO GmbH, Germany) and is flowed continuously through an outer side passage of the air cooler. The temperature of the outlet is adjusted using the temperature setting in the circulator.

The humid-air nozzle was fabricated from a polyacetal tube (8 mm inner diameter, 10 mm outer diameter). To prevent the influence of ambient air, the measurement sample is inserted into the nozzle tube 10 mm from its edge. In addition, two holes were created in the nozzle wall at the sample position. A 5 mm-diameter hole is used as a path for incident X-rays and to monitor the sample using a co-axial CCD camera. A second, 7 mm-diameter hole at the opposite side acts as a path for diffraction up to 1.67 Å (horizontal) and 1.32 Å (vertical) resolution at a wavelength of 1 Å [Figs. 1 ▸(*b*) and 1 ▸(*c*), respectively]. The outlet temperature, *t*_2_ (°C), is measured inside the humid-air nozzle using a T-type thermocouple, which is made of copper–constantan and is used for low-temperature applications such as cryogenics in crystallography.

It was necessary to estimate the outlet RH (*h*_2_), since installing a humidity sensor at the outlet caused disturbed flow and therefore could not be done. At temperature *t* (in °C), the density of saturated water vapour calculated using the August–Roche–Magnus formula (Alduchov & Eskridge, 1996 ▸) is as follows:
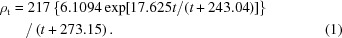
Here, the volumetric humidity ρ_t_ (g m^−3^) was used as the humidity unit, and RH was calculated as the ratio of the volumetric humidity of the air against that of saturated air. Then, the outlet RH, *h*_2_, was calculated using the volumetric humidity of saturated air at the temperature *t*_1_ of mixed air from the humidifier, ρ_t__1_, its RH, *h*_1_, for the humidity adjustment unit, and the volumetric humidity of saturated air at outlet temperature *t*_2_, ρ_t__2_, for the humid-air nozzle. The mixing ratio of dry and saturated-humidity gases was calculated using the following formula:
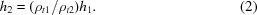



The digital mass-flow controller for each gas was operated using the *LabVIEW* software (National Instruments Corporation, USA) on a Microsoft Windows PC. The total flow rate was set to 3 l min^−1^. The outlet humidity was controlled through the flow rates of dry and saturated humid gases by manual operation or the proportional-integral-differential (PID) control provided by *LabVIEW*. The outlet temperature was manually controlled by the circulator temperature using *EasyTEMP* (JULABO GmbH) through the RS232 interface. Therefore, usually, the outlet temperature was fixed during experiments, but the outlet humidity was maintained even when the circulator temperature was changed. All logs from the wired sensors were stored to a PC from a multi-data logger.

To evaluate the humidifier’s performance, we measured the stability of the blowing gas by PID control (Table 1 ▸). Temperature and humidity were recorded every 10 s for 30 min. The average and standard deviation (SD) were calculated using these data.

### 2.2. Protein-crystal-handling workbench for low temperature   

The experimental environment around protein crystals should be maintained at the desired temperature while they are being collected. Gloveboxes are commonly used to maintain the experimental environment; however, most gloveboxes are not temperature controllable and are unsuitable for delicate work with bare hands. For such experiments, we developed a temperature-controlled airflow workbench, TeC-W [Fig. 1 ▸(*d*)]. The airstream is supplied by an N_2_ gas generator (KOFLOC Corporation Ltd) and is cooled using a coolant chilled by the F50HE refrigerated–heating circulator (JULABO GmbH) with an HE-25-025T2 multi-tubular heat exchanger (Fuji Industry Co. Ltd, Japan). The dry N_2_ gas is charged into the chamber of the workbench to create a positive pressure inside it. The chamber has a large inner volume of 0.36 m^3^ (0.9 m height, 0.8 m width, 0.5 m depth) and leaks because of two holes which allow insertion of bare hands into the chamber. Therefore, the airstream was supplied at a greater flow rate of 15 l min^−1^, and the chamber itself was also cooled by the coolant flowing in the wall piping of the chamber’s insulation panel. However, the workbench does not control humidity.

### 2.3. Evaluation of the TeCH-1 humidifier using the HAG method   

#### 2.3.1. Sample preparation   

To evaluate a low-temperature environment using the HAG method, we used CcO crystals in a carbon monoxide-bound reduced state. The crystals belong to space group *P*2_1_2_1_2_1_ and were obtained at 4°C, as described previously (Mochizuki *et al.*, 1999 ▸). Since CcO crystals are sensitive to temperature changes, all operations with them were conducted at 4°C. The crystals in the carbon monoxide-bound reduced state were prepared from resting oxidized CcO crystals obtained from CcO purified from bovine hearts (Muramoto *et al.*, 2010 ▸). Briefly, crystallized resting oxidized samples were harvested before diffraction experiments into a solution with an increased concentration of 21%(*w*/*w*) ethyl­ene glycol (EG) and 4.5%(*w*/*w*) polyethyl­ene glycol 4000 (PEG 4000) against the crystallization solution. The samples were then incubated for 30 min in a reductant solution composed of 40 m*M* sodium phosphate (pH 6.1), 21%(*w*/*w*) EG, 4.5%(*w*/*w*) PEG 4000, 0.2%(*w*/*v*) decyl­maltoside and 5 m*M* di­thio­nite saturated with carbon monoxide. To confirm the carbon monoxide-bound reduced state, we analysed the absorbance spectrum of each CcO crystal before it was coated with glue (Shimada *et al.*, 2017 ▸).

CcO crystal mounting via the HAG method was performed using the TeC-W workbench at 4°C. An aqueous glue solution consisting of 5%(*w*/*v*) polyvinyl alcohol (PVA8000; Japan VAM & POVAL Co. Ltd, Japan), 2%(*w*/*v*) EG and 1%(*w*/*v*) PEG 4000 was used. The glue solution, a LithoLoops crystal mounting loop (Protein Wave Corporation, Japan) with a diameter larger than the CcO crystals, and a Cryo Tong (Hampton Research Corporation) were all cooled to 4°C in the workbench before use. The CcO crystals were coated with the glue, and a small amount of the glue solution was also applied to and spread over the crystal mounting loop. Each crystal was fished up directly from the harvesting solution by the glue-coated loop. Then, the CcO crystal mounted in the loop was clamped with the Cryo Tong in the workbench. During transportation of the CcO crystals to the goniometer, the temperature of the specimen needs to be maintained, especially in low-temperature experiments. Therefore, after the clamped crystal had been taken out of the workbench, it was covered with a 4°C thermal gel, which was allowed to set on the diffractometer. Immediately after setting, the humid-air nozzle was moved to the sample position.

#### 2.3.2. Diffraction data collection   

To measure diffraction, the TeCH-1 humidifier was installed on the SPring-8 BL38B1 beamline, together with a one-axis-rotation goniometer (Kohzu Seiki Co. Ltd, Japan), a Rayonix MX225HE detector and a nitro­gen Cryostream system GN2 (Rigaku Corporation, Japan). At the experimental station of the beamline, RT was rigorously maintained at 21°C, with a temperature stability of ±0.3°C h^−1^, while the humidity was broadly kept at 40–60%RH. The target temperature of the humidity-controlled air was set at 4°C for all diffraction experiments.

**Figure 2:**
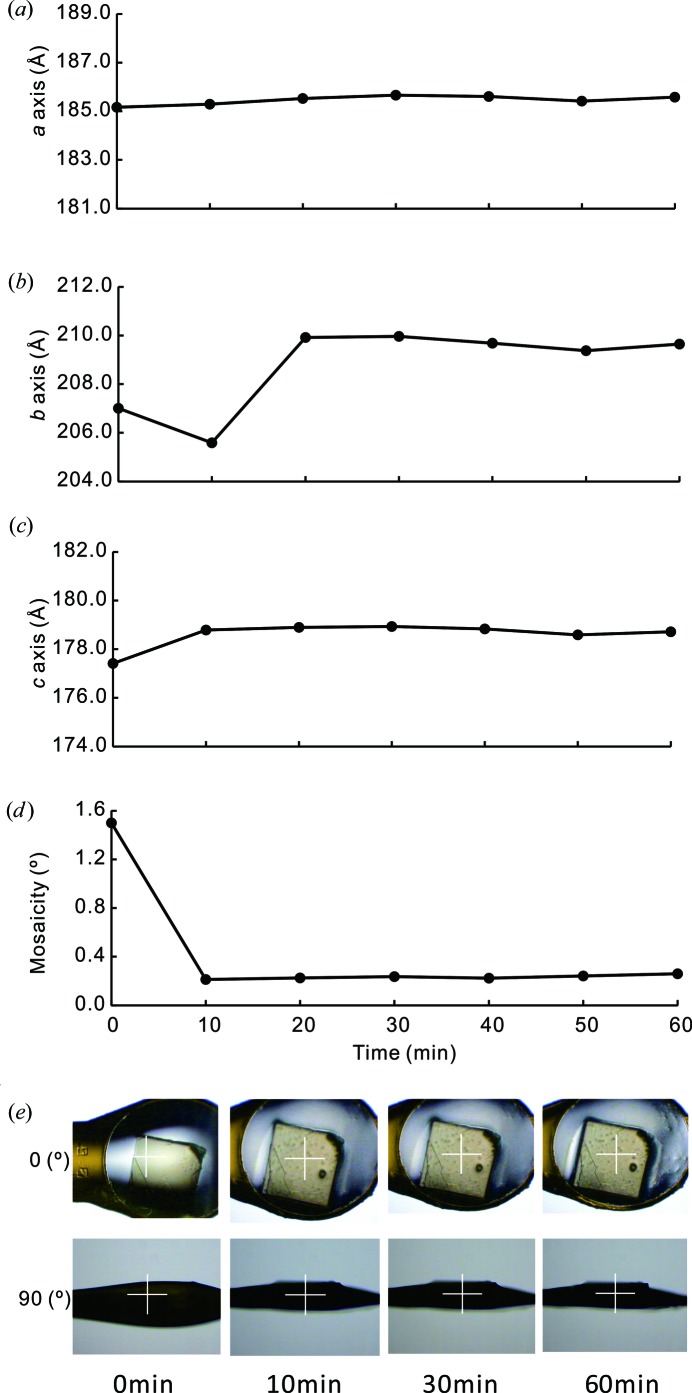
Time course of lattice transformation of a CcO crystal subjected to a constant RH of >98%RH at 4°C. Plots show the crystallographic *a* axis (*a*), *b* axis (*b*), *c* axis (*c*) and mosaicity (*d*). (*e*) The mounted crystal shown at 0 and 90° using a co-axis camera. Each arm of the white centre cross is 400 µm.

To evaluate CcO crystal quality during the diffraction experiment, the mosaicity and unit-cell parameters for each diffraction image were processed using the *HKL2000* suite (Otwinowski & Minor, 1997 ▸). The conditions of the diffraction experiment were set as follows: the exposure time and rotation angle per image were 2 s and 1°, respectively; the incident beam was irradiated perpendicular to the *ac* plane of the CcO crystals [as shown in Fig. 2 ▸(*e*) upper row]. The beam size was 200 µm (height) × 100 µm (width) at the sample position with a photon flux of 9.5 × 10^10^ photons s^−1^ at a wavelength of 1 Å.

## 3. Results and discussion   

The TeCH-1 humidifier with the F50HE refrigerated–heating circulator successfully provided humid gas at a wide range of temperatures and humidity levels: 4–20°C and 20–99.5%RH, respectively. When using the F32HE refrigerated–heating circulator, the humidity SD tended to increase with higher humidity at 4°C, and the PID control did not work stably. The results suggested that improving the heat-insulating performance and the heat-exchange efficiency of the air cooler would allow more precise temperature and humidity control. The newly designed humid-air nozzle was also important for maintaining samples. When using the previous humid-air nozzle, which was similar to those of conventional cryostreams, the CcO crystal quality was not maintained, even at low temperatures. To avoid interfusion of ambient air into outlet air, insertion of CcO crystals into the humid-air nozzle, as described before [Fig. 1 ▸(*c*)], was successful. Meanwhile, the low-temperature TeC-W workbench worked well to maintain the temperature within 4.0 ± 0.2°C. During CcO crystal-handing with bare hands, the temperature of the TeC-W workbench increased by 0.5°C.

By combined use of the two devices, we successfully applied the low-temperature HAG method to CcO crystals. At RT, the CcO crystals became mechanically friable, and the diffraction quality decreased for the 5–10 min required for the crystal-handling operation (Fig. S1). However, the CcO crystals maintained mechanical rigidity when kept at 4°C using the TeC-W workbench. Therefore, we could expect to obtain better diffraction data at ambient temperature and reveal side-chain conformations clearly. Previously, a data set of 2.2 Å resolution was collected at 6.85°C using capillary mounting (Muramoto *et al.*, 2010 ▸). This data set might be better compared with the present study. However, the two data sets are not directly comparable because the former data set was collected using the higher-flux undulator SPring-8 BL44XU beamline and nine crystals with large crystal sizes (PDB code 3ag1). Considering these results, we set the target resolution as ∼2.5 Å.

To determine the optimum humidity for CcO crystals, we performed three trials at 4°C. First, we investigated temporal changes of lattice constants of the CcO crystals to evaluate the equilibration time in response to environmental changes in the CcO crystals under the almost saturated humidity of >98%RH by manual flow operation without PID control. This humidity value was the maximum without dew condensation inside the humidifier. Diffraction images were collected every 10 min for 60 min. It took ∼10 min for the lattice constants to reach equilibrium, and the lattices were then stably maintained for over 1 h (Fig. 2 ▸). During the first hour, the glue around the CcO crystal gradually thinned because of dehydration [Fig. 2 ▸(*e*)]. We previously reported a similar relaxation time and long-term crystal stability in tetragonal lysozyme crystals at RT (Baba *et al.*, 2013 ▸).

Next, we decreased the humidity from 98%RH to determine the optimum humidity without PID control. A new CcO crystal was mounted, and we waited for 5 min after relaxation. Then we gradually decreased the humidity in 0.5–1% steps after each time delay of 2–3 min. The CcO crystal diffraction quality evaluated by crystal mosaicity and the resolution of the observed Bragg reflections remained intact at 96%RH or higher, but at 95%RH, the CcO crystal diffraction degraded in 5 min. The damage was not recovered with either rehydration to 96%RH or exposure to >99%RH.

**Figure 3:**
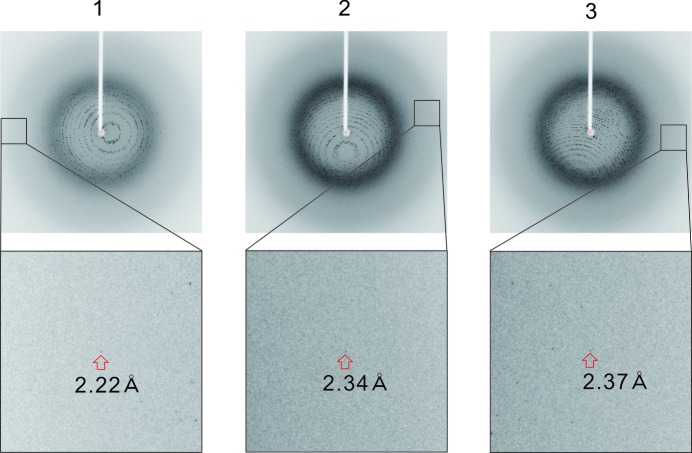
Diffraction images of three CcO crystals using the HAG method at 4°C and 98%RH.

Finally, to improve the reproducibility of the humidity-control experiments, we examined PID control by comparison with lattice constants of three CcO crystals after relaxation under an optimum humidity of 98%RH. Under these conditions (Fig. 3 ▸), the lattice constants of the three CcO crystals were close to each other (Table 2 ▸). This result and our empirical observations suggested that the isomorphism and diffraction quality of CcO crystals can be better maintained using the low-temperature HAG method than by using the conventional method. This low-temperature HAG method is quite useful for obtaining data sets by merging data sets from a number of CcO crystals.

## 4. Conclusions   

The TeCH-1 humidifier and TeC-W workbench successfully controlled temperature between 4 and 20°C. Using the combined device, we were able to perform a diffraction experiment on CcO crystals which were temperature controlled for crystal-handling operations and humidity controlled under the crystallization temperature. Because the HAG method has proved adaptable for protein crystals under various crystallization conditions, we intend to attempt to improve the performance of the TeCH-1 humidifier over a wider temperature range in future. In this study, we expanded the HAG method to low-temperature experiments.

## Supplementary Material

To explain crystal damage at room temperature. DOI: 10.1107/S1600576719006435/ei5041sup1.pdf

## Figures and Tables

**Table 1:** Stability of temperature and humidity at the humidifier outlet

	Target temperature (°C)/humidity (%RH)				
	4/80	10/80	15/80	20/80	4/98
Average temperature (°C)	3.77 (0.12)	9.88 (0.15)	15.13 (0.13)	19.75 (0.11)	3.78 (0.13)
	4.10 (0.07)	9.95 (0.07)	15.13 (0.07)	19.93 (0.08)	4.09 (0.07)
Average humidity (%RH)	80.03 (0.65)	80.01 (0.77)	79.98 (0.65)	81.10 (0.52)	98.02 (0.81)
	79.98 (0.49)	80.02 (0.40)	80.03 (0.41)	80.06 (0.35)	97.97 (0.55)

The upper and lower values in each column correspond to the device equipped with F32HE and F50HE refrigerated–heating circulators, respectively. Values in parentheses are standard deviations.

**Table 2:** Unit-cell parameters and mosaicities of three CcO crystals at 4°C and 98%RH

Crystal No.	1	2	3
Unit cell			
*a* (Å)	184.81	184.73	184.72
*b* (Å)	208.77	208.65	208.68
*c* (Å)	178.64	178.47	178.54
Apparent resolution (Å)	2.22	2.34	2.37
Mosaicity (°)	0.099	0.159	0.269

The apparent resolution is the maximum resolution spot with index processing (σ cut-off 5.0) performed via *HKL2000*.
