# Supplementary material for: A temperature-controlled cold-gas humidifier and its application to protein crystals with the humid-air and glue-coating method
Source: J Appl Crystallogr. 2019 Jun 14;52(Pt 4):699–705. doi: 10.1107/S1600576719006435 (PMC6662993; doi:10.1107/S1600576719006435)
Supplement: Supplementary file 1 [file j-52-00699-sup1.pdf]

JOURNAL OF APPLIED CRYSTALLOGRAPHY

Volume 52 (2019)

Supporting information for article:

A temperature-controlled cold gas humidifier and its application to protein crystals with the HAG method

Seiki Baba, Atsuhiro Shimada, Nobuhiro Mizuno, Junpei Baba, Hideo Ago, Masaki Yamamoto and Takashi Kumasaka

Supporting information

In the previous experiment (PDB ID 3AG1), CcO crystals were packaged into a glass capillary tube in a 4°C cold room. To confirm the effect of temperature, A CcO crystal was manipulated using the TeC-W workbench at 4°C and maintained in 98%RH at RT using the original humidifier (Baba *et al.*, 2013) during the diffraction experiment. The maximum resolution of the first image was 2.6 Å (Fig. S1a). However, after 10 min, the resolution decreased to 3.5 Å (Fig. S1b). This result suggested that CcO crystal at RT have decreased quality.

Figure S1 Diffraction images of CcO crystal using the HAG method at RT and 98%RH. The CcO crystal was manipulated at 4°C using the TeC-W workbench. (a) The first diffraction image immediately taken after mounting on a diffractometer. (b) The second diffraction image after 10 min. CcO, cytochrome c oxidase; HAG, humid-air and glue-coating; RT, room temperature; RH, relative humidity.

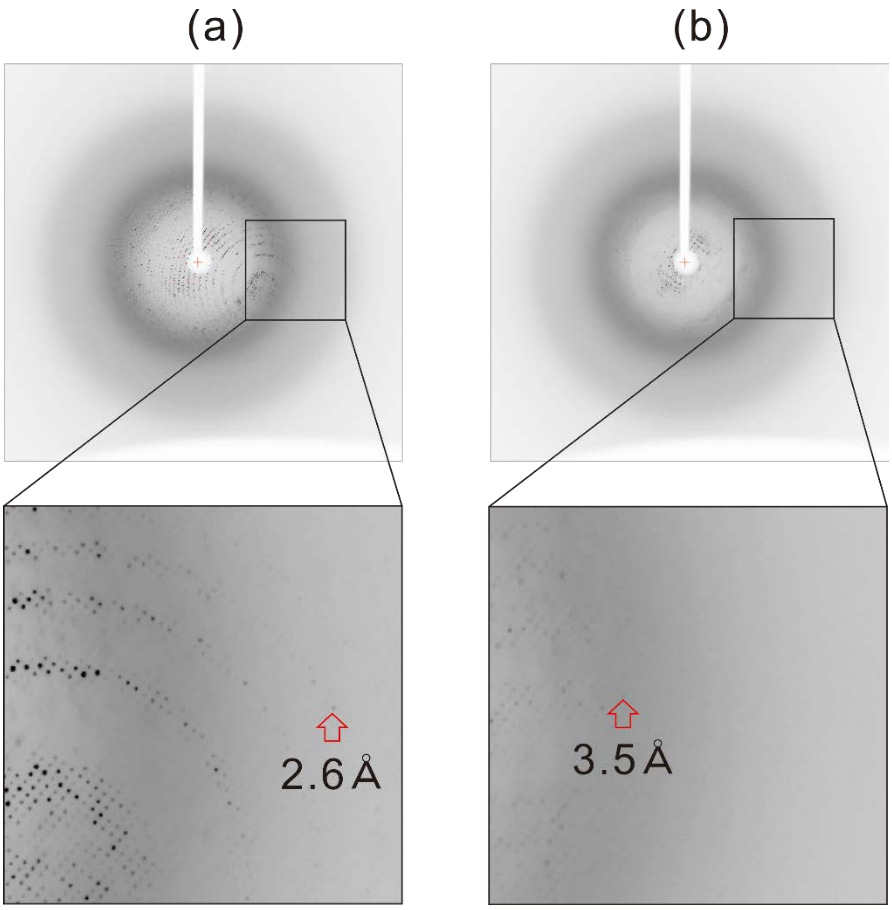

IMPORTANT: this document contains embedded data - to preserve data integrity, please ensure where possible that the IUCr Word tools (available from http://journals.iucr.org/services/docxtemplate/) are installed when editing this document.
